# Treatment of Pott’s Curvature

**Published:** 1897-04

**Authors:** 


### TREATMENT OF POTT'S CURVATURE.

Calot (*Sem. Med.*, Dec. 23, 1896), judging from his experience with thirty-seven children suffering from this disease, states that all children with Pott's curvature can be cured without deformity. His method of procedure is first to anesthetize the patient, then to forcibly straighten the spine by pulling the upper and lower portions backward, at the same time making strong pressure on the convexity of the curve. After straightening, a plaster jacket is applied reaching from the head to the pelvis.

In cases in which it is impossible to correct the deformity by these means, the projecting spinal processes are removed. The author states that in two out of his thirty-seven cases it was necessary to excise the posterior wedge of bone which prevented the vertebral column from straightening. Only five to ten months are needed for a cure, instead of two or three years under the usual treatment. The occurrence of paralysis is also largely prevented by this method.

Calot showed five children before the Academy of Medicine, whose humps, after existing for six months to six years, had been treated by this method. In some there was no trace of the curvature, in others but very little of the deformity remained.—*Modern Medicine.*

